# Environmental Enrichment in Kennelled Pit Bull Terriers (*Canis lupus familiaris*)

**DOI:** 10.3390/ani7040027

**Published:** 2017-03-23

**Authors:** Jenna Kiddie, Anna Bodymore, Alex Dittrich

**Affiliations:** Anglia Ruskin University, East Road, Cambridge CB1 1PT, UK; abodymore@yahoo.com (A.B.); Alex.Dittrich@anglia.ac.uk (A.D.)

**Keywords:** dog behaviour, dog welfare, environmental enrichment, Pit Bull Terrier, shelter welfare

## Abstract

**Simple Summary:**

Rescue shelters, although set-up with good intentions, may lead to poor welfare of the animals that they house, especially when the shelter organisation is under-resourced. This study therefore investigated the behavioural effects of cheap and locally accessible objects added to the cages of rescued Pit Bull Terrier type dogs in the Philippines to determine whether it is possible for shelter organisations with limited funds and staffing to improve the welfare of their dogs with minimal additional pressure on their time and financial budgets. The addition of enrichment (coconuts; cardboard bed; or cardboard partition) to the dogs’ kennels positively affected their behavior but no one treatment was better at achieving this result. The results therefore suggest that it is possible for under-resourced rescue shelters to use cheap, locally available objects to improve the welfare of dogs in their care.

**Abstract:**

Although social enrichment can be considered beneficial in helping dogs cope with the kennel environment, when taking individual needs into account, it places a large demand on the carers and may not be appropriate in under-resourced kennels. Some kennels are also designed in such a way that there is too much social interaction, in that individuals cannot choose to distance themselves from conspecifics. This study therefore aimed to assess the effects of easily accessible enrichment on the behaviour of kennelled Pit Bull Terrier type dogs rescued from a dog-fighting ring in the Philippines. Thirty-six dogs were allocated to one of three treatment groups following a matched-subject design: (i) cardboard bed provision; (ii) coconut provision; and (iii) visual contact with dogs housed in adjacent cages obstructed with cardboard partitions. Behavioural diversity and the duration and frequency of individual behaviours were analysed using linear mixed-effect models. Yawning frequencies and time spent lying down and sitting decreased during treatment. No particular treatment was more influential in these behavioural changes. In conclusion, enrichment, regardless of type, affected the dogs’ behaviour, with some effects depending on the sex of the dogs. Therefore, it is possible to cheaply and sustainably enrich the lives of dogs living in highly constrained environments, however, further research is required to refine the methods used.

## 1. Introduction

Despite their popularity as pets and their valued utility as working and sport animals many dogs end up living in government or charity run kennels [1,2,3]. These dogs may be pets or retired working or sport dogs relinquished by their owners; but they may also be abandoned or lost pets; long-term stray or feral dogs; and individual abuse cases or banned types seized by local authorities or welfare charities. Dogs are also often rescued in large numbers in attempts to control roaming dog populations, after natural disasters, or from situations of willful neglect and violence where their welfare is considered to be severely compromised, for example, from activities relating to the dog-meat trade and dog-fighting. As large-scale rescues are often unexpected or occur in low socioeconomic areas, welfare charities can sometimes be underprepared for such large numbers of dogs in terms of facilities and resources, which can in turn compromise the welfare of the dogs they have rescued. For example, such rescues can place dogs in overcrowded and inappropriate environments [4], conditions that are likely to exacerbate the acute and chronic stress experienced by dogs entering better resourced kennels; kennel environments, for practical and financial reasons, are usually restricted in size and complexity and may offer only limited environmental and social stimulation [5]. Social and spatial restriction [6,7], and too little or too much environmental variability [8,9] can lead to chronic stress with accompanying psychological distress, fear and frustration, a restricted ability to perform species-specific behaviours, increased levels of aggression [5], and therefore potentially physical injury from fighting. Crowding, insufficient resources, a lack of handler knowledge, training and equipment can all exacerbate these sources of stress and increase susceptibility to disease [4].

An increasingly common method of reducing stress in captive animals is environmental enrichment, which describes the adaptation of captive environments for the benefit of the inhabitants [10]. The goals of environmental enrichment are to: increase behavioural choices and encourage species appropriate behaviours in order to enhance welfare (behavioural diversity); reduce abnormal behavior; normalise temporal behavioural patterns; increase positive utilisation of the environment; and increase the ability to cope with challenges in a more normal way [11]. Social, or animate, enrichment involves the introduction of additional human [12] or intra-specific [13] interaction, whereas, physical, or inanimate, enrichment may take the form of inanimate objects, for example toys or cage furniture [7], feeding enrichment [14], or olfactory [15] and auditory [16] stimulation. Although social enrichment has been found to be highly beneficial for kennelled dogs [7,12,13] it does place a larger demand on the time of the carers and may not be appropriate in under-resourced kennels. Therefore, cheap and easily accessible or easily made physical enrichment may be more practical and feasible for many rescue charities. The needs of the animals should be considered alongside locally available materials. For example, Pullen et al. [17] found that dogs prefer toys that they can chew and that are presented on the floor, as opposed to hanging. Commercially available toys can be expensive, therefore local substitutes that are safe for dogs to chew, such as coconuts, should be considered, after risk has been assessed [18]. The provision of bunk beds has been found to positively affect welfare [19] but in cages that are already raised off the ground, comfortable bedding may ease the effects of prolonged lying on a hard surface, which can lead to pressure-related hyperkeratotic areas on pressure points [20]. Similarly, beds and bedding can be expensive and many materials are too easily shredded or soiled by dogs and therefore require frequent replacement or cleaning. Cardboard is an easily accessible material that can be replaced at minimal cost when soiled or damaged. Providing a privacy screen may also offer kennelled dogs a greater degree of choice, allowing the dogs some control over visual contact with other dogs and kennel staff [7,20]. This can easily be achieved by strategically attaching cardboard or sacking over part of the external surface of the kennel housing.

Any changes to animal housing should be monitored and evaluated [21]. Welfare can be assessed by making inferences from behavioural measures [22] and in contrast to physiological measures of welfare, behavioural measures are relatively practical, inexpensive and non-invasive, with some measurements only requiring observation from a distance [23], which is more feasible for understaffed facilities. For example, time budgets measure how animals allocate their time and can be used by animal care staff to assess the impact of changes to management practices or changes to the physical or social environment [24]. Behavioural diversity can be calculated from activity budgets and is an indication of behavioural opportunity and degree of control. For example, less behavioural diversity is normally exhibited by captive animals than by wild animals [25] but it can be increased in captivity by improving the quality of housing [26], which has been achieved by using feeding enrichment in captive-born small wild cats [27] and red foxes [28]. Behavioural diversity has also more recently been investigated in kennelled dogs and was found to increase with quality of life [29] and novelty [30]. It is calculated using the Shannon Diversity Index, which is the product of the total number of behaviours in the population and the relative frequency of each behaviour in the sample [27].

This study therefore aimed to use behavioural measures to assess the effect of cheap and sustainable environmental enrichment on the welfare of kennelled Pit Bull Terriers that had been rescued from a dog-fighting ring in the Philippines. Dogs given coconuts were hypothesised to perform increased durations and frequencies of normal and active behaviours [31], such as walking and chewing enrichment, and behavioural diversity [27], whereas the dogs given cardboard beds and cardboard partitions were expected to show more relaxed behaviours [7,20], such as sitting or lying down for longer durations. All groups were hypothesized to show less distress-related behaviours such as yawning, panting and licking the cage [31].

## 2. Materials and Methods

### 2.1. Animals and Housing

Thirty-six neutered, adult dogs (>3 years old) were selected from a shelter population of 160 dogs in the Philippines: four aggressive dogs were excluded from the initial sample selection for safety reasons, the remaining dogs were assigned a number, sex-matched and pseudo-randomly selected using a random number generator [32]. This prevented choosing dogs based on appearance or temperament. All the dogs were Pit Bull Terriers or Pit Bull Terrier types that had been rescued from a fighting ring 2.5 years previously. The dogs were observed in their own cages and were therefore interspersed with dogs that were not being observed in the study. The dogs were individually housed in wire cages that were raised off the ground, with plastic mesh flooring. Standard cages had a floor area of 90 cm × 120 cm and a height of 90 cm in front sloping to 97.5 cm at the back. Two males were housed in double cages, which were made up of two standard cages welded back to back, with a floor area of 90 cm × 240 cm and a height of 90 cm in front sloping to 97.5 cm at the back. All cages were 75 cm from ground level, raised on metal legs. The cages were arranged in straight lines, with adjacent cages positioned approximately 30 cm apart. Some cages were facing another row of cages that were approximately 1m away. The cages were located outside but covered with a shower proof material for protection against the rain and sun. The dogs had constant access to a water bowl, which was also used to give them their once-daily meal. This meal consisted of boiled rice and vegetables, and a small quantity of kibble. Some dogs were occasionally given coconuts to chew. The dogs were walked on average once monthly, but most were given weekly access to an outdoor paddock. One of the dogs was removed from the study after the first day due to ill health. He was replaced with another randomly selected male dog from the initial sample.

### 2.2. Experimental Procedure

This study was conducted in November 2014. The dogs were pseudo-randomly (matched for sex) and equally allocated to one of three mutually exclusive enrichment groups: partitions, whole brown coconuts, and cardboard beds (6 females and 6 males in each group). All but one dog’s partitions were made from cardboard, hung on the outside of the two side walls of the cage in order to reduce visual contact with the dogs in adjacent cages. One of the female dog’s partitions had to be made from plastic sacking as that cage was more susceptible to rain and the cardboard disintegrated. Partitions were chosen to reduce visual contact with adjacent dogs in order to reduce overstimulation, while maintaining visual contact with dogs in opposing cages. The cardboard beds were unfolded cardboard boxes, placed inside the cage, affording the dogs a potentially more comfortable surface than the plastic mesh flooring. Whole brown coconuts were provided for dogs to play with and chew on or rip open.

The experimental procedure consisted of four phases: habituation to cameras placed outside kennels and to the researcher’s presence (over 4 days); baseline—the dogs were filmed without enrichment (over 4 days); treatment–enrichment items were provided on alternate days (over 7 days); and post-exposure–the enrichment was no longer provided (over 4 days). Enrichment was provided after feeding and cleaning and just prior to video-recording commencing. Dogs were video recorded for 30 min, between 9.30 am and 12.30 pm (once feeding and cleaning were finished, which varied from day to day and between dogs), on the first and last day of the baseline and post-exposure phases and on alternate days during the treatment phase (recording coincided with the days of enrichment provision); behavioural footage was not collected during the habituation phase. The average temperature was 31.5 °C and the average humidity was 81% over the course of the study; however, this data was taken from published local records and not from the immediate surrounds of the dog kennels, therefore this data has not been used in any analysis.

### 2.3. Data Analysis

Behavioural data was analysed from the videos using a freeware program called Behavioural Observation Research Interactive Software (BORIS) [33]. An ethogram (Table 1) was generated from watching a small subsample of videos, this was done after the data was collected for logistical reasons. State and event behaviours were recorded using continuous sampling. Interaction with the different enrichment objects was recorded but not analysed for the partition group as most of the dogs in this group were not able to interact with their enrichment; lying on the cardboard bed and chewing the bed or the coconut were combined and were only analysed in relation to treatment day and sex.

Behavioural diversity was calculated per dog per study day using the Shannon diversity index. The index was calculated using the following equation [27]:

$$H=\sum p_{i}log(\frac{1}{p_{i}})$$

where *p_i_* is the proportion of time engaged in the *i*th behaviour. Larger values of H indicate greater diversity.

### 2.4. Statistical Analysis

The cardboard beds were generally shredded by the dogs, only one female dog used the cardboard sheet as a bed, therefore as the bedding was utilised in a similar fashion to the coconuts, data from these two groups were analysed as one group: interactive enrichment group. Therefore, a new hypothesis was formed before analysis, that the dogs in the new interactive enrichment group would show greater behavioural diversity and more active behaviours than dogs given cardboard partitions.

Behavioural diversity and 12 individual behaviour duration percentages and frequencies (Table 1) from the first and last day of each phase were analysed using linear mixed-effect models (normal distribution with an identity link Gaussian error structure), treatment group and sex were fitted as fixed-effect factors, dog fitted as a random factor, and day and phase fitted as repeated measures. Variables were further analysed using reduced models, initially with treatment removed as a fixed factor, and then only with phase fitted as a fixed-effect factor and dog as a random factor. Full and reduced models were compared to assess goodness of fit using the Akaike information criteria (AIC) values. Interaction with enrichment objects was similarly analysed with sex and treatment day (only for the two treatment days with enrichment provision) fitted as fixed-effect factors and dog as a random factor. All analyses were carried out using SPSS (IBM SPSS Statistics 20, IBM, Chicago, IL, USA).

### 2.5. Ethical Approval

This study was approved by the Animal and Environmental Biology Group’s Departmental Research Ethics Panel at Anglia Ruskin University (Project Identification Code: A&EB DREP14-020).

## 3. Results

**Figure 1:**
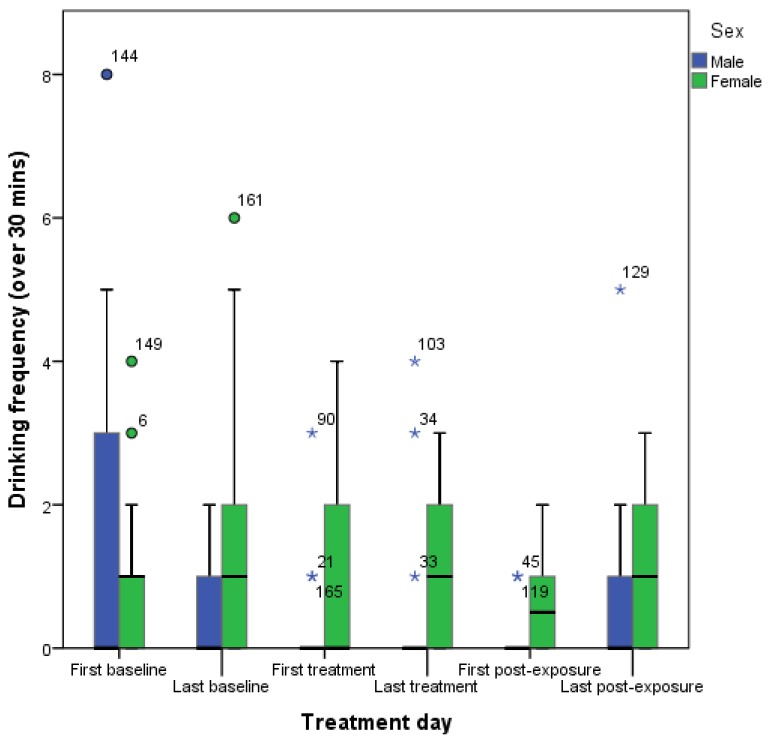
Box plot of drinking frequency, over a 30 min period, of male (*n* = 18) and female (*n* = 18) Pit Bull Terriers before, during and after exposure to environmental enrichment. Circles and asterisks represent outliers with adjacent case numbers.

Only one full linear mixed effect model (with treatment group and sex fitted as fixed-effect factors, dog fitted as a random factor, and day and phase fitted as repeated measures) was significant (F(2, 206) = 5.072, *p* = 0.007, AIC = 660.742): there was a statistically significant association between drinking frequency and the sex of the dog (F(1, 206) = 10.089, *p* = 0.002). However, treatment had no effect (F(1, 206) = 0.053, *p* = 0.818), showing no significant difference between the interactive physical enrichment and the partition provisions, and was so removed from the model. The resulting reduced model had a lower AIC value (659.42) and was therefore an improvement on the previous model: females were found to drink more than males (F(1, 207) = 10.423, *p* = 0.001). Moreover, subject had an effect on drinking frequency (*Z* = 1.965, *p* = 0.049). Finally, all interactions between treatment phase and day also showed statistical significance (*Z* < 4, *p* < 0.01). Females drank more than males in general but males drank more on the first baseline day than females, and more than they drank on any other day of the study (Figure 1).

**Figure 2:**
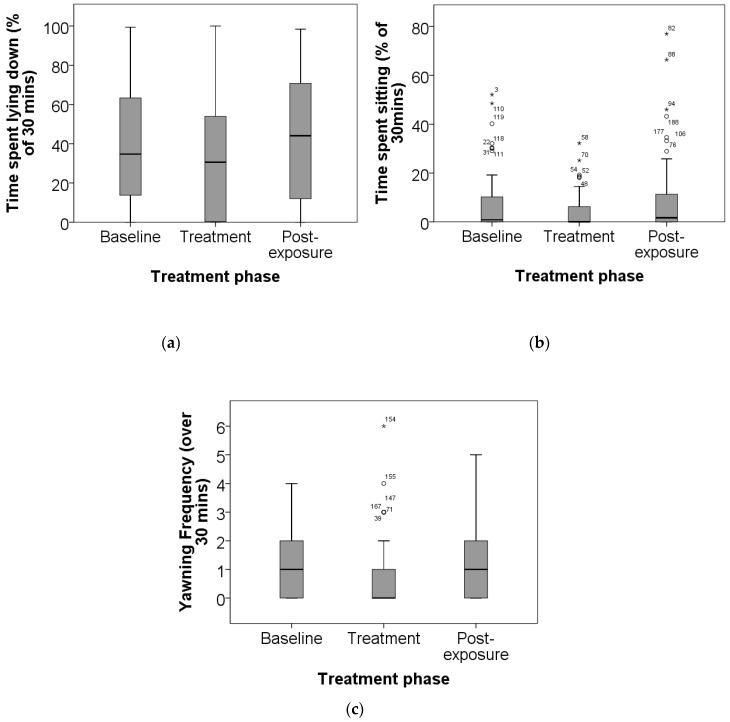
The proportion of time spent (**a**) lying down; (**b**) sitting down; and (**c**) the yawning frequency of Pit Bull Terriers (18 males and 18 females) before, during and after exposure to environmental enrichment. Note. Different axes are used for the two duration behaviour box plots. Circles and asterisks represent outliers with adjacent case numbers.

Variables were further analysed with phase fitted as a fixed-effect factor and dog as a random factor. Three of these models were significant and assessed to have better goodness of fit than the full models: lying; sitting; and yawning differed significantly between phases, with varying degrees of individual variation. Dogs spent less time lying down during the treatment phase (F(2, 206) = 3.20, *p* = 0.043) (Figure 2a), although there was significant individual variation (*Z* = 3.29, *p* = 0.001). Dogs also spent less time sitting down during the treatment phase (F(2, 206) = 4.35, *p* = 0.014) (Figure 2b), also with significant individual variation (*Z* = 2.9, *p* = 0.004). Dogs also yawned less in the treatment phase (F(2, 206) = 4.99, *p* = 0.008) (Figure 2c), but there was no individual effect (*Z* = 1.73, *p* = 0.085). The duration and frequencies of all behaviours are presented in Table 2.

## 4. Discussion

This study aimed to assess the effect of cheap and sustainable enrichment on the welfare of kennelled Pit Bull Terriers in the Philippines. Dogs given coconuts were hypothesised to perform increased durations and frequencies of active behaviours, such as walking and chewing enrichment, and also increased behavioural diversity, whereas the dogs given cardboard beds and cardboard partitions were expected to show more relaxed behaviours, such as sitting or lying down for longer durations. All groups were hypothesized to show less distress-related behaviours such as yawning and panting. However, the dogs did not utilise all of the enrichment provisions in the way intended and in fact, the types of enrichment did not appear to differ significantly in their effects on the behavioural measures investigated in this study. Most notably, the cardboard beds were shredded by all but one of the female dogs, therefore treating them as chewable or manipulable objects rather than comfortable bedding. This is not surprising given the otherwise barren cage environment these dogs were living in. Dogs have been found to prefer toys that they can chew [17] and therefore appear to find this activity reinforcing. The need for these particular dogs to chew on objects may have been enhanced by the lack of hard food in their diet, although this was not a focus of the study. Therefore, the provision of cardboard as bedding may have been more successful if the same dogs had also been given coconuts to chew. The dogs given cardboard beds and coconuts were consequently grouped together for analysis as the interaction with the enrichment objects was very similar. A new hypothesis was therefore formed before analysis, that these dogs would show greater behavioural diversity and more active behaviours than dogs given cardboard partitions. However, the types of enrichment did not differ significantly in their effects on the behavioural measures utilised in this study.

As hypothesized time spent lying and sitting decreased in the cardboard bed and coconut combined group during the treatment phase as these dogs were actively engaging with their enrichment objects, thus supporting the hypothesis that these objects were enriching. Conversely, time spent lying down and sitting was hypothesised to be higher in the cardboard partition group than in the cardboard bed and coconut combined group due to increased control over social interactions [7] leading to reduced social stimulation, allowing dogs to relax more. However, there was no statistically significant difference between the treatment groups. Instead, there was an overall decrease in lying down and sitting during the treatment phase, in line with the other treatment group. This may be because that although the dogs had more privacy and could theoretically exhibit more relaxed behaviours they may actually have valued the undisturbed visual contact with all neighbouring dogs [34] despite their dog fighting history. Alternatively, the discomfort likely caused by the abrasive plastic mesh flooring may have deterred dogs from sitting or lying down. Provision of partitions in conjunction with a comfortable bed may therefore also be more appropriate and warrants further study. There was however significant individual variation in these two behaviours suggesting that dogs responded to different stressors in their environments, i.e., either the enrichment or the flooring/other sources of chronic stress, or that they responded differently to the same stressors, i.e., they had different coping styles [35]. From a scientific perspective, this result supports the importance of including the individual as a factor in statistical analysis, but from a welfare perspective it is important to tailor welfare assessments and interventions to the individual.

The decrease in yawning frequencies during the treatment phase was expected. Yawning is associated with chronic stress in dogs [36]. It is considered a displacement behaviour, exhibited in contexts of conflict [37], and may act to reduce arousal [38]. This has been suggested by studies on ACTH-induced behavioural syndromes, which include displacement behaviours such as yawning and grooming [38,39]. The decrease in yawning frequency during the treatment phase adds further support to the conclusion that the treatment is enriching.

The changes in drinking frequency of males was much higher on the first baseline day than the second baseline day, which had similar levels to the remaining experimental days, with and without enrichment. This suggests that the males may have needed a slightly longer habituation period as it may be that they were still reacting to the presence of the cameras and the researcher on this first day of data collection. Females were also found to drink more than males on the remaining experimental days; this is a common pattern found in young and adult people from many industrial countries [40] and may therefore reflect a sex difference in general hydration requirements. Alternatively, this result may reflect sex-dependent differences in coping with chronic environmental stress, although findings that female dogs are less able to cope are inconsistent across studies [35,41]. This result therefore requires further study.

Barking and kennel rearing were not significantly associated with any of the parameters studied, although these could be considered distress-related behaviours and therefore hypothesised to decrease in frequency during the treatment phase. It is therefore likely that these behaviours were more influenced by external factors [7], such as the presence of caretakers [42] or social facilitation [43], than the treatment conditions of the experiment.

Grooming, panting and urinating were also not found to be significantly associated with treatment. Increased grooming, panting and urinating have previously been found to be associated with chronic stress (e.g., [6,35,36,44]), however, grooming has also been associated with anticipation and positive welfare [22]. Therefore individual variation may have influenced these behaviours more than the treatment.

Despite the apparent enriching effects of the chosen objects, there are limits to the practicality of using cardboard. As the cages were not completely sheltered from the rain, and because the caretakers used hosed water to clean the pens, the shredded cardboard became very wet and moulded to the mesh flooring, which was very difficult and time-consuming to remove once dried. Of course the cardboard could be temporarily removed during cleaning but this again adds to caretaker time and is not a practical option for dogs that respond aggressively to the caretakers leaning into their cage. This last point of course raises questions relating to the welfare and ethical concerns regarding the keeping of aggressive dogs in housing set-ups, such as cages, where the dogs cannot be separated from care staff for cleaning and feeding purposes. Therefore, more robust materials should be considered for cheap and sustainable bedding for practical use.

## 5. Conclusions

In conclusion, enrichment, regardless of type, had some effect on the dogs’ behaviour, with some effects depending on the sex and the individual. Therefore, it is possible to cheaply and sustainably enrich the lives of dogs living in highly constrained environments, however, further research is required to refine the methods used.

## Figures and Tables

**Table 1:** Ethogram used to calculate the activity budget of the kennelled dogs.

Behaviour	Description
Lie	Part of both upper and lower body in contact with the bottom of the cage.
Sit	Hindquarters in contact with the bottom of the cage, front legs extended.
Interact with coconut or cardboard bed	Part of both upper and lower body in contact with cardboard bed, or chewing the coconut or cardboard.
Stand	Standing with all four paws in contact with the bottom of the cage.
Panting	Breathing deeply and quickly with mouth open.
Barking	Vocalisation, one or a series of short sharp sounds.
Yawn	Opens mouth wide and closes eyes.
Playing with bowl *	Interacting with water bowl, pushing, picking up.
Chewing cage *	Chewing any part of the cage.
Licking cage *	Licking any part of the cage.
Drink	Laps water.
Autogroom	Licks or chews part of own body.
Defecate *	Squats to defecate.
Urinate	Squats or cocks leg to urinate.
Walk	Ambulates at any speed.
Kennel rear	Stands on hind legs with forelegs against side of kennel.
Other	Behaviours that are not defined, especially coprophagy.

N.B. * denotes behaviours that were not included in the analysis due to homogeneity in the data.

**Table 2:** Means and standard errors of all behaviours on each treatment day analysed.

Day	Mean/SE	Sit	Walk	Stand	Lie	Groom	Pant	Bark	Urinate	Drink	Kennel Rear	Yawn	Interact with Coconut/Bed
First baseline	Mean	9.37	8.47	22.01	38.69	4.10	8.45	41.03	0.31	1.28	4.69	1.34	0.00
	SE	2.85	1.91	3.06	5.81	1.19	2.35	15.42	0.10	0.36	3.80	0.24	0.00
Last baseline	Mean	6.31	7.20	22.76	41.83	1.90	12.70	69.03	0.33	0.94	2.81	1.28	0.00
	SE	1.62	1.35	2.41	4.80	0.89	2.61	27.41	0.09	0.24	1.79	0.27	0.00
First treatment	Mean	2.98	4.76	22.58	36.33	2.31	11.32	36.44	0.28	0.61	1.97	0.64	22.89
	SE	0.88	1.33	2.61	4.84	0.77	2.07	19.41	0.09	0.18	1.44	0.23	3.58
Last treatment	Mean	4.94	7.41	30.15	28.45	1.27	8.71	79.72	0.44	0.75	3.97	0.75	13.81
	SE	1.26	1.59	3.52	5.01	0.54	2.24	29.05	0.11	0.20	2.39	0.15	3.91
First post-exposure	Mean	11.82	5.19	24.75	45.08	2.36	4.32	41.92	0.33	0.42	1.00	1.14	0.00
	SE	3.09	0.96	3.61	5.79	0.69	1.63	17.60	0.11	0.12	0.62	0.23	0.00
Last post-exposure	Mean	5.73	8.43	26.29	38.57	1.29	11.22	88.08	0.67	0.83	2.39	1.06	0.00
	SE	1.69	1.60	3.36	5.02	0.45	2.55	19.27	0.12	0.19	0.88	0.19	0.00
Total	Mean	6.78	6.86	24.85	38.14	2.14	9.49	59.99	0.40	0.79	2.74	1.02	6.20
	SE	0.83	0.60	1.28	2.13	0.31	0.93	9.13	0.04	0.09	0.79	0.09	1.17
